# Sotagliflozin Decreases Postprandial Glucose and Insulin Concentrations by Delaying Intestinal Glucose Absorption

**DOI:** 10.1210/clinem/dgz258

**Published:** 2019-12-14

**Authors:** David R Powell, Brian Zambrowicz, Linda Morrow, Carine Beysen, Marcus Hompesch, Scott Turner, Marc Hellerstein, Phillip Banks, Paul Strumph, Pablo Lapuerta

**Affiliations:** 1 Lexicon Pharmaceuticals, Inc, The Woodlands, Texas; 2 ProSciento, Inc, Chula Vista, California; 3 Pliant Therapeutics, South San Francisco, California; 4 University of California, Berkeley, California; 5 Metavant Sciences, Ltd., Durham, North Carolina

**Keywords:** Sotagliflozin, type 2 diabetes, SGLT2 inhibitor, SGLT1 inhibitor, intestinal glucose absorption, postprandial glucose

## Abstract

**Context:**

The effect of sotagliflozin (a dual sodium–glucose cotransporter [SGLT] 2 and SGLT1 inhibitor) on intestinal glucose absorption has not been investigated in humans.

**Objective:**

To measure rate of appearance of oral glucose (R_a_O) using a dual glucose tracer method following standardized mixed meals taken after single sotagliflozin or canagliflozin doses.

**Setting:**

Clinical research organization

**Design and participants:**

In a double-blind, 3-period crossover study (NCT01916863), 24 healthy participants were randomized to 2 cohorts of 12 participants. Within each cohort, participants were randomly assigned single oral doses of either sotagliflozin 400 mg, canagliflozin 300 mg, or placebo on each of test days 1, 8, and 15. On test days, Cohort 1 had breakfast containing [6,6-^2^H_2_] glucose 0.25 hours postdose and lunch containing [1-^2^H_1_] glucose 5.25 hours postdose; Cohort 2 had breakfast containing no labeled glucose 0.25 hours postdose and lunch containing [6,6-^2^H_2_] glucose 4.25 hours postdose. All participants received a 10- to 15-hour continuous [U-^13^C_6_] glucose infusion starting 5 hours before their first [6,6-^2^H_2_] glucose-containing meal.

**Main Outcome:**

R_a_O, postprandial glucose (PPG), and postprandial insulin.

**Results:**

Sotagliflozin and canagliflozin decreased area under the curve (AUC)_0–1 hour_ and/or AUC_0–2 hours_ for R_a_O, PPG, and insulin after breakfast and/or the 4.25-hour postdose lunch (*P *< .05 versus placebo). After the 5.25-hour postdose lunch, sotagliflozin lowered R_a_O AUC_0–1 hour_ and PPG AUC_0–5 hours_ versus both placebo and canagliflozin (*P* < .05).

**Conclusions:**

Sotagliflozin delayed and blunted intestinal glucose absorption after meals, resulting in lower PPG and insulin levels, likely due to prolonged local inhibition of intestinal SGLT1 that persisted for ≥5 hours after dosing.

Sodium–glucose cotransporter (SGLT) 2 is primarily responsible for the reabsorption of filtered glucose from the lumen of the renal proximal tubule, while SGLT1 is responsible for the absorption of oral glucose from the lumen of the small intestine (1,2). Orally available small-molecule SGLT2 inhibitors block renal glucose reabsorption, resulting in increased urinary glucose excretion (UGE) that is associated with significant decreases in glycated hemoglobin (A1C) levels, body weight, and blood pressure in patients with type 2 diabetes (T2D) (3,4). In patients with T2D and high cardiovascular risk, SGLT2 inhibitor treatment significantly lowers the risk of cardiovascular events (5,6) and slows the progression of chronic kidney disease toward renal failure (7,8). Patients with type 1 diabetes (T1D) also respond to SGLT2 inhibitors with significantly lower A1C levels, body weight, and blood pressure, and with an increased percentage of time that their blood glucose levels are within the acceptable range of 70 to 180 mg/dL, namely the time in range (TIR) (9–17).

Sotagliflozin is a dual inhibitor of SGLT2 (median inhibitory concentration [IC_50_] = 1.8 nM) and SGLT1 (IC_50_ = 36 nM) (18). In humans, sotagliflozin improves glycemic control partly by increasing UGE via renal SGLT2 inhibition and partly by decreasing incremental postprandial glucose (PPG) excursions after an oral glucose challenge (18,19). This decrease in PPG is likely due to sotagliflozin-mediated inhibition of intestinal SGLT1, resulting in a delayed rate of appearance of oral glucose (R_a_O). In animals, the sotagliflozin-mediated decrease in gastric inhibitory polypeptide (GIP) levels and increases in glucagon-like peptide-1 (GLP-1) and peptide YY (PYY) levels after oral glucose challenge are consistent with local inhibition of intestinal SGLT1-mediated glucose absorption (20–22). However, the effect of sotagliflozin on R_a_O has not yet been studied in humans.

Canagliflozin, an SGLT2 inhibitor (IC_50_ = 4.2 nM) that also inhibits SGLT1 (IC_50_ = 663 nM) with much lower potency than sotagliflozin, also decreases incremental PPG excursions during the first meal after a dose of more than 200 mg (3,23–26). In healthy adults, a single oral 300-mg canagliflozin dose significantly decreased PPG after a glucose-containing meal that started 0.5 hours after dosing, but had no effect on PPG after meals that started 4.5 or 10.5 hours after dosing (24). The decrease in incremental PPG following canagliflozin given 20 minutes before a standard glucose-containing meal was primarily due to delayed and blunted R_a_O rather than increased UGE (23,26). Canagliflozin also delayed and blunted the incremental PPG excursion relative to dapagliflozin (a more selective inhibitor of SGLT2 [IC_50_ = 1.2 nM] than SGLT1 [IC_50_ = 1400 nM]), which had no effect on PPG (3,25). Together, these studies suggested that blunting of incremental PPG by canagliflozin was due to delayed and blunted R_a_O mediated by inhibition of intestinal SGLT1.

These data suggest that sotagliflozin should effectively delay and blunt R_a_O. To test this hypothesis, we performed a 3-period crossover study that included a dual-isotope tracer protocol to compare the effects of single doses of placebo, 400 mg sotagliflozin and 300 mg canagliflozin on PPG fluxes and other parameters in healthy adults.

## Methods

### Study design and participants

This single-center, partially double-blind, randomized, single-dose, 3-period crossover study was conducted from August 6, 2013, to October 14, 2013, at ProSciento Inc, Chula Vista, CA, USA, to assess R_a_O in blood (referred to as intestinal glucose absorption) using a dual glucose tracer method after single doses of sotagliflozin or canagliflozin in healthy adults.

**Figure 1.**
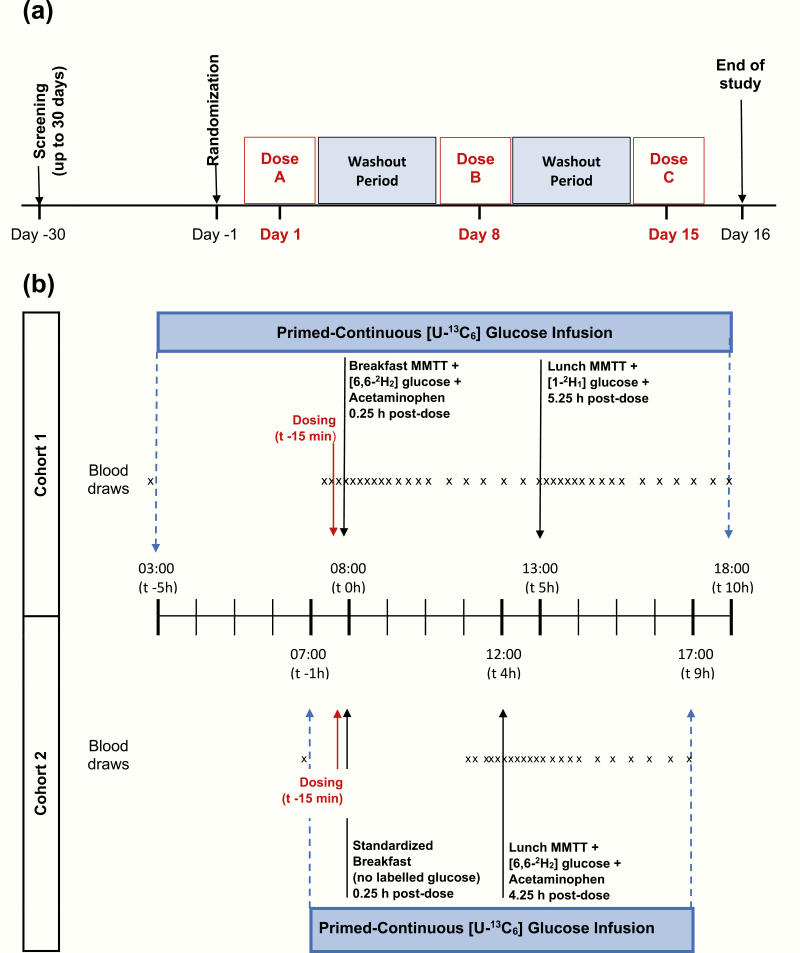
Schematic of (A) overall study design and (B) stable isotope, blood collection and meal protocol design for Cohort 1 (top panel) and Cohort 2 (bottom panel).

Twenty-four eligible males and females were randomly assigned to 2 cohorts of 12 participants each. Within each cohort, participants were randomly assigned to receive single oral doses of either 400 mg sotagliflozin, 300 mg canagliflozin (INVOKANA®), or sotagliflozin-identical placebo on each of 3 successive treatment days separated by 7-day washout periods: on days 1, 8, and 15, respectively (Fig. 1A). The 3 treatment sequences, to which the participants, investigators, and sponsors were blinded, were generated to satisfy requirements of a Williams Latin square design. Each cohort was assigned different stable isotope and meal protocols to enable analysis of glucose absorption after breakfast and lunch on each treatment day. For Cohort 1, the dual glucose tracer method was used with both the breakfast and the lunch mixed meal tolerance tests (MMTTs), while for Cohort 2 the dual glucose tracer method was used in combination with a lunch MMTT only (Fig. 1B). This study was partially double-blind because subjects and investigators were blinded to dosing of sotagliflozin versus placebo, but not to dosing of canagliflozin.

Eligible participants were aged 18 to 55 years and had normal blood pressure, heart rate and electrocardiogram, kidney and liver function, and a body mass index of 18 to 35 kg/m^2^. Exclusion criteria included receipt of any protein or antibody-based therapeutic agents within 3 months before screening; prior exposure to sotagliflozin or canagliflozin; and any surgical or medical condition that might have interfered with the absorption, distribution, metabolism, or excretion of study treatment.

All participants provided written informed consent before enrollment. The protocol and amendment were reviewed by a centralized Institutional Review Board. The study was conducted in accordance with Good Clinical Practice as required by the International Council on Harmonisation guidelines and in accordance with US laws and the ethical principles of the Declaration of Helsinki.

### Procedures and assessments

Before each treatment day, all participants fasted overnight for a minimum of 10 hours, then took their assigned study drug orally with water approximately 15 minutes before breakfast (Fig. 1B). The breakfast and lunch MMTTs had identical nutrient compositions (50% kcal carbohydrates, 33% kcal fat, and 17% kcal protein) and consisted of a drink containing 75 g of glucose, boiled egg (70 g), and mozzarella string cheese (70 g). Part of the glucose in the drink was replaced with stable isotope-labeled glucose for the dual-tracer assessments (described further below). Breakfast was taken 0.25 hours postdose in both cohorts; lunch was taken 5.25 hours postdose by Cohort 1 and 4.25 hours postdose by Cohort 2. All meals had to be consumed within 20 minutes, but without undue haste.

In Cohort 1, intestinal glucose absorption was assessed following breakfast and lunch MMTTs. To distinguish between glucose sources from breakfast and lunch, different stable isotope-labeled glucoses were administered with the breakfast (15 g of the 75 g of total glucose was replaced with [6,6-^2^H_2_] glucose) and lunch (8 g of the 75 g of total glucose was replaced with [1-^2^H_1_] glucose) MMTTs (Fig. 1B). In Cohort 2, glucose fluxes were assessed only after lunch to avoid any potential confounding effects from breakfast. In this cohort’s lunch MMTT, 15 g of the 75 g of glucose was replaced with [6,6-^2^H_2_] glucose. Participants in both cohorts also received a continuous [U-^13^C_6_] glucose infusion starting 5 hours before their first [6,6-^2^H_2_] glucose-containing MMTT. The [U-^13^C_6_] glucose infusion was continued until the last blood sample to measure glucose fluxes had been collected.

Glucose fluxes during the MMTT were determined from the measured plasma glucose concentration and plasma [U-^13^C_6_] glucose, [6,6-^2^H_2_] glucose, and [1-^2^H_1_] glucose enrichment profiles using the dual-isotope tracer method. Glucose fluxes were calculated using nonsteady state equations of Steele as previously described (27) and a total distribution volume of 160 mL/kg. The rate of appearance of total glucose in blood (R_a_T; labeled and unlabeled glucose from infused, ingested, and endogenous glucose sources) was calculated from the infused glucose tracer ([U-^13^C_6_] glucose). R_a_O was calculated from the rate of appearance of ingested glucose tracer [6,6-^2^H_2_] glucose or [1-^2^H_1_] glucose, and the glucose tracer enrichment of the meals. Endogenous glucose production (EGP) was calculated by subtracting R_a_O from the rate of appearance of total glucose (R_a_T), and total glucose disposal (R_d_T) was calculated by subtracting the change in glucose mass from R_a_T.

Acetaminophen absorption was used as an indirect measure of gastric emptying. Liquid acetaminophen (1000 mg) was administered immediately after the first MMTT on all treatment days (Fig. 1B) and blood acetaminophen concentrations were then measured over the subsequent 5 hours.

Blood samples were collected before the start of the stable isotope infusions and then every 10 to 30 minutes during the 10 hours after breakfast in Cohort 1, and during the 5 hours after lunch in Cohort 2, to measure plasma glucose enrichments to calculate glucose fluxes (R_a_O, R_a_T, R_d_T, and EGP rates). Plasma glucose enrichments were analyzed from deproteinized plasma and converted to the aldonitrile penta-acetate derivative for gas chromatography–mass spectrometry analysis. During MMTTs containing [6,6-^2^H_2_] glucose, many of the blood samples were used to measure glucose by enzymatic assay, insulin and connecting peptide (C-peptide) by electrochemiluminescence, active GLP-1 (aGLP-1) and total GLP-1 (tGLP-1) by electrochemiluminescent immunoassay, total PYY and GIP by enzyme-linked immunosorbent assay, and glucagon by radioimmunoassay (all performed by Pacific Biomarkers, Seattle, WA); in addition, acetaminophen was measured by enzymatic assay (Labcorp, Burlington, NC). The 24-hour urine collections were obtained to evaluate UGE for each treatment period.

### Objectives and endpoints

The primary objective was to evaluate the effect of single doses of sotagliflozin or canagliflozin on R_a_O following breakfast (Cohort 1) and lunch (Cohort 2) MMTTs in healthy adults. The primary endpoint was the total area under the (rate–time) curve over the intervals of 0 to 1 hour (AUC_0–1 hour_) and 0 to 2 hours (AUC_0–2 hours_) for R_a_O for both the breakfast (Cohort 1) and lunch (Cohort 2) MMTTs.

Secondary objectives and endpoints that applied to both breakfast of Cohort 1 and lunch of Cohort 2 were to evaluate the effects of single doses of sotagliflozin and canagliflozin on PPG (total and incremental AUC_0–4 hours_); insulin and C-peptide (total and incremental AUC_0–4 hours_); R_a_T (total AUC_0–5 hours_); R_d_T (total AUC_0–5 hours_); EGP (total AUC_0–5 hours_); and tGLP-1, aGLP-1, PYY, GIP, and glucagon (total and incremental AUC_0–4 hours_). Other secondary endpoints included PPG for Cohort 1 during the lunch MMTT (total and incremental AUC_5–10 hours_) and UGE for Cohorts 1 and 2 (grams/24 hours).

Exploratory objectives included the effects of single doses of sotagliflozin and canagliflozin on gastric emptying rate (total AUC_0–5 hours_); R_a_O (AUC_0–5 hours_ and AUC_2–5 hours_); R_a_O (AUC_5–10 hours_) for the lunch MMTT of Cohort 1; and PPG, insulin, C-peptide, tGLP-1, aGLP-1, PYY, GIP, glucagon, and gastric emptying (AUC_0–1 hour_ and AUC_0–2 hours_). For Cohort 1, PPG and R_a_O AUC_5–10 hours_ were equivalent to, and presented as, Cohort 1 lunch AUC_0–5 hours_ so that for all MMTTs 0 hour represented the time of meal initiation.

### Statistical analyses

Continuous variables were summarized descriptively. Categorical variables were summarized descriptively by their participant counts and associated percentages. For the primary pharmacodynamic parameters of R_a_O AUC_0–1 hours_ and R_a_O AUC_0–2 hours_, data were log-transformed for analysis. A linear mixed effects model, which included fixed effects for treatment, sequence, and treatment period with a random effect for participant nested within the sequence, was used to test treatment differences for the study endpoints within each meal test group (cohort). Separate models were used for each cohort. Least squares mean differences with 2-sided 90% confidence intervals were calculated for canagliflozin versus placebo, sotagliflozin versus canagliflozin, and sotagliflozin versus placebo. Statistical assessment of treatment differences for the primary endpoint comparisons of each active drug versus placebo involved 1-sided tests with α = .05. Tests between the 2 active arms and for all nonprimary endpoint comparisons were based on 2-sided statistics. Point estimates of treatment effects used maximum likelihood methods. Tests of treatment effects for the primary and secondary endpoints are prespecified. Due to the exploratory nature of the study, adjustments for multiplicity were not performed. Hence, nominal *P* values and confidence intervals were provided as descriptive statistical summaries of the statistical data. Interpretation of pharmacodynamic effects due to findings from active study treatment comparisons should largely be based on the point estimates of effect and the confidence intervals derived from these point estimates; *P* values may be useful to guide inferences.

## Results

### Study participants

The demographic characteristics of the 24 study participants who were enrolled and randomly assigned into the 2 cohorts are summarized in Table 1; they were well balanced between the 2 groups.

**Table 1.** Participants’ demographic characteristics at baseline.

Demographic	Cohort 1 (n = 12)	Cohort 2 (n = 12)
Mean age (SD), years	37.1 (11.6)	43.3 (9.2)
Sex		
Male	11 (91.7)	11 (91.7)
Female	1 (8.3)	1 (8.3)
Race		
Black or African American	5 (41.7)	5 (41.7)
White	7 (58.3)	8^*a*^ (66.7)
Mean weight (SD), kg	91.1 (11.5)	92.0 (10.6)
Mean body mass index (SD), kg/m^2^	29.7 (4.2)	29.5 (2.8)

Data are presented as (%) unless stated otherwise.

^*a*^Some patients indicated more than one race.

### Rate of appearance of oral glucose

**Figure 2.**
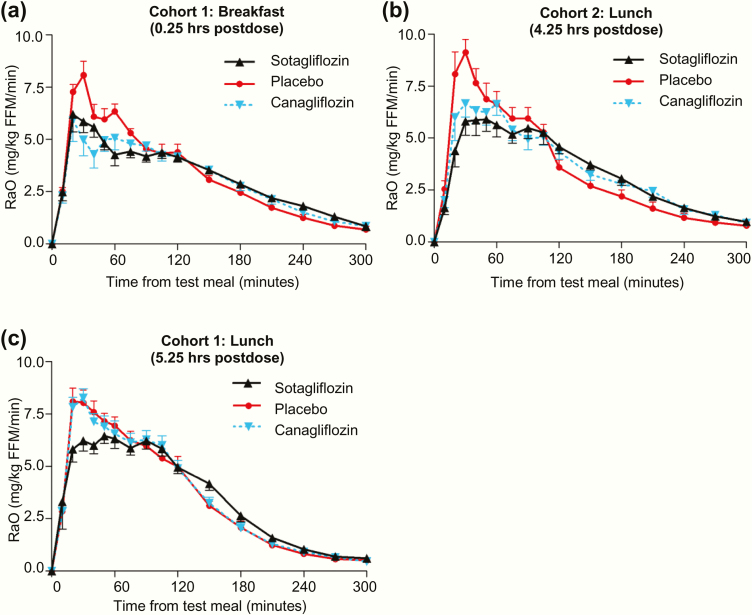
Effect of single doses of sotagliflozin or canagliflozin on the rate of appearance of oral glucose (R_a_O) in blood during the mixed meal tolerance tests (MMTTs). All data points are mean ± standard error of the mean.

**Figure 3.**
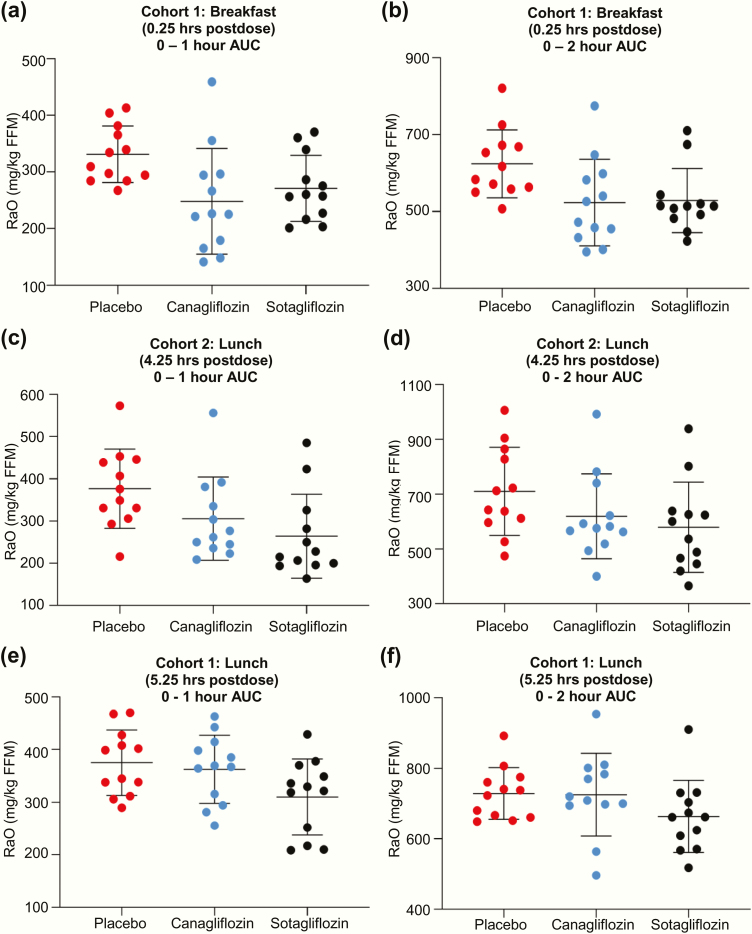
Individual subject data for the effect of single doses of sotagliflozin or canagliflozin on R_a_O AUC_0–1 hour_ and R_a_O AUC_0–2 hours_ during the MMTTs. Data are presented as mean ± SD.

R_a_O data are presented in Table 2, with timeline data presented in Fig. 2 and interindividual variability data presented in Fig.3. After the breakfast MMTT starting 0.25 hours postdose (Fig. 2A and Fig. 3A and 3B), both sotagliflozin and canagliflozin delayed and blunted R_a_O compared with placebo: the AUC_0–1 hour_ decreased an insignificant 19% with sotagliflozin (*P* = .06) and 29% with canagliflozin (*P* = .005), while R_a_O AUC_0–2 hours_ decreased 15% with sotagliflozin (*P* = .01) and 17% with canagliflozin (*P* = .005). In contrast, R_a_O AUC_2–5 hours_ increased 20% with sotagliflozin (*P* = .003) and 14% with canagliflozin (*P* = .023); this compensated for the decrease in R_a_O over the first 2 hours, resulting in insignificant R_a_O AUC_0–5 hours_ decreases of 3% for sotagliflozin (*P* = .31) and 5% for canagliflozin (*P* = .05), all relative to placebo.

**Table 2.** R_a_O measured following MMTTs started 0.25, 4.25, and 5.25 hours after single oral doses of sotagliflozin, canagliflozin, or placebo.

		AUC (mg/kg FFM), LSM			LSMR (90% CI of LSMR), *P*^*a*^					
MMTT	Interval (hours)	Pbo	Sota	Cana	Sota vs. Pbo	*P*	Cana vs. Pbo	*P*	Sota vs, Cana	*P*
Cohort 1 Breakfast0.25 hours postdose	0–1	328	265	233	0.81 (0.67, 0.98)	.06	0.71 (0.59, 0.86)	**.005**	1.14 (0.94, 1.37)	0.25
	0–2	619	523	513	0.85 (0.76, 0.94)	**.01**	0.83 (0.75, 0.92)	**.005**	1.02 (0.92, 1.13)	0.75
	2–5	350	420	400	1.20 (1.09, 1.32)	**.003**	1.14 (1.04, 1.25)	**.023**	1.05 (0.96, 1.15)	0.36
	0–5	975	950	926	0.97 (0.93, 1.02)	.31	0.95 (0.91, 0.99)	.05	1.03 (0.98, 1.07)	**0.31**
Cohort 2 Lunch 4.25 hours postdose	0–1	366	250	294	0.68 (0.62, 0.75)	**<.001**	0.80 (0.73, 0.89)	**<.001**	0.85 (0.77, 0.94)	**0.01**
	0–2	695	560	604	0.81 (0.74, 0.88)	**<.001**	0.87 (0.79, 0.95)	**.014**	0.93 (0.85, 1.02)	0.17
	2–5	297	432	406	1.45 (1.29, 1.63)	**<.001**	1.37 (1.22, 1.54)	**<.001**	1.06 (0.95, 1.19)	0.37
	0–5	1023	1007	1030	0.98 (0.95, 1.02)	.39	1.01 (0.8, 1.04)	.73	0.98 (0.95, 1.01)	0.23
Cohort 1 Lunch 5.25 hours postdose	0–1	370	302	357	0.81 (0.72, 0.93)	**.013**	0.96 (0.85, 1.10)	.63	0.84 (0.74, 0.96)	**0.036**
	0–2	726	657	716	0.90 (0.82, 1.00)	.09	0.99 (0.90, 1.09)	.81	0.92 (0.83, 1.01)	0.14
	2–5	313	375	306	1.23 (1.05, 1.43)	**.033**	1.02 (0.88, 1.19)	.81	1.20 (1.03, 1.40)	0.05
	0–5	1040	1045	1045	1.00 (0.97, 1.04)	.82	1.00 (0.97, 1.04)	.83	1.00 (0.97, 1.03)	1

Abbreviations: Cana, canagliflozin; FFM, fat-free mass; LSM, geometric least square mean; LSMR, geometric LSM ratio (Sota vs Pbo, Cana vs Pbo, Sota vs Cana); MMTT, mixed meal tolerance test; Pbo, placebo; R_a_O, rate of appearance of oral glucose; Sota, sotagliflozin. All *P* values < .05 are in bold.

^*a*^ *P* value based on null hypothesis that LSMR = 1.

After the lunch MMTT starting 4.25 hours postdose (Fig. 2B and Fig. 3C and 3D), R_a_O AUC_0–1 hour_ decreased 32% with sotagliflozin (*P* < .001) and 20% with canagliflozin (*P* < .001) relative to placebo, with sotagliflozin showing a greater decrease relative to canagliflozin (*P* = .01), while R_a_O AUC_0–2 hours_ decreased with both sotagliflozin (19%, *P* < .001) and canagliflozin (13%, *P* = .014) relative to placebo. R_a_O AUC_2–5 hours_ again increased with sotagliflozin (45%, *P* < .001) and canagliflozin (37%, *P* < .001), resulting in R_a_O AUC_0–5 hours_ that was not significantly different between either compound and placebo.

After the lunch MMTT starting 5.25 hours postdose (Fig. 2C and Fig. 3E and 3F), R_a_O AUC_0–5 hours_ did not differ significantly between either compound and placebo. A post hoc analysis, performed to evaluate the same AUC time points as the first 2 MMTTs, showed that R_a_O AUC_0–1 hour_ decreased 19% for sotagliflozin (*P* = .013) but only 4% for canagliflozin (*P* = .63), relative to placebo. Also, the decrease in R_a_O AUC_0–1 hour_ was greater with sotagliflozin relative to canagliflozin (*P* = .036). R_a_O AUC_2–5 hours_ was increased 23% by sotagliflozin (*P* = .033), but only 2% by canagliflozin (*P* = .81), relative to placebo, providing further evidence that sotagliflozin, but not canagliflozin, delayed R_a_O during this MMTT.

### Postprandial glucose concentrations

**Figure 4.**
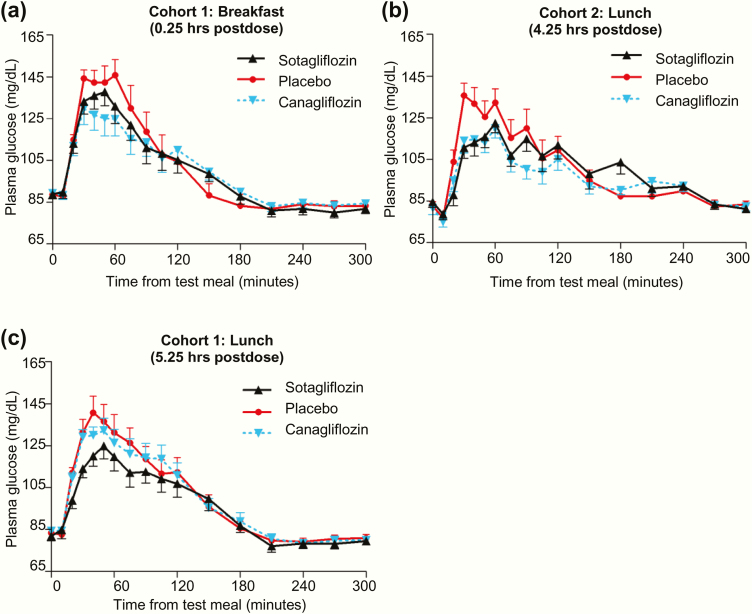
Effect of single doses of sotagliflozin or canagliflozin on postprandial glucose (PPG) concentrations during the MMTTs. All data points are mean ± standard error of the mean.

Both sotagliflozin and canagliflozin blunted the increase in PPG concentrations after meals in a manner that mirrored their effect on R_a_O (Fig. 4 and Table 3). After breakfast (Fig. 4A), incremental PPG AUC_0–1 hour_ and AUC_0–2 hours_ showed insignificant decreases of 25% (*P* = .20 and *P* = .14, respectively) for sotagliflozin and decreases of 43% (*P* = .016) and 36% (*P* = .026), respectively, for canagliflozin, all relative to placebo. Consistent with these results, total PPG AUC_0–1 hour_ and AUC_0–2 hours_ decreased significantly with canagliflozin relative to placebo (*P* = .026 and *P* = .036, respectively).

**Table 3.** Total and incremental PPG measured following MMTTs started 0.25, 4.25, and 5.25 hours after single oral doses of sotagliflozin, canagliflozin, or placebo.

		AUC (h×mg/dL), LSM			LSMR (90% CI of LSMR), *P* value^a^					
Variable, MMTT	Interval (hours)	Pbo	Sota	Cana	Sota vs. Pbo	*P*	Cana vs. Pbo	*P*	Sota vs. Cana	*P*
**PPG total**	0–1	130	121	117	0.92 (0.85, 1.00)	.10	0.90 (0.83, 0.97)	**.026**	1.03 (0.95, 1.11)	.52
Cohort 1, Breakfast	0–2	255	237	233	0.93 (0.87, 1.00)	.08	0.91 (0.85, 0.98)	**.036**	1.02 (0.95, 1.09)	.67
0.25 hours postdose	0–4	442	423	428	0.96 (0.91, 1.01)	.14	0.97 (0.92, 1.02)	.26	0.99 (0.94, 1.04)	.71
**PPG incremental**	0–1	41	31	23	0.75 (0.52, 1.09)	.2	0.57 (0.39, 0.82)	**.016**	1.32 (0.92, 1.91)	.2
Cohort 1, Breakfast	0–2	75	56	48	0.75 (0.55, 1.04)	.14	0.64 (0.47, 0.88)	**.026**	1.18 (0.86, 1.62)	.39
0.25 hours postdose	0–4	88	69	64	0.78 (0.57, 1.07)	.19	0.73 (0.54, 1.00)	.1	1.07 (0.78, 1.46)	.73
**PPG total**	0–1	121	106	107	0.88 (0.84, 0.93)	**<.001**	0.88 (0.84, 0.93)	**<.001**	1.00 (0.95, 1.05)	.89
Cohort 2, Lunch	0–2	240	222	213	0.92 (0.87, 0.98)	**.044**	0.89 (0.83, 0.95)	**.004**	1.04 (0.98, 1.11)	.29
4.25 hours postdose	0–4	439	425	410	0.97 (0.92, 1.02)	.31	0.93 (0.89, 0.99)	**.044**	1.04 (0.98, 1.09)	.28
**PPG Incremental**	0–1	37	20	24	0.53 (0.41, 0.69)	**<.001**	0.64 (0.49, 0.83)	**.009**	0.83 (0.63, 1.08)	.23
Cohort 2, Lunch	0–2	70	48	49	0.69 (0.52, 0.91)	**.033**	0.70 (0.53, 0.93)	**.044**	0.98 (0.74, 1.30)	.89
4.25 hours postdose	0–4	100	77	80	0.77 (0.57, 1.05)	.16	0.80 (0.59, 1.10)	.24	0.96 (0.70, 1.30)	.81
**PPG total**	0–1	118	106	115	0.90 (0.85, 0.96)	**.007**	0.97 (0.92, 1.03)	.44	0.93 (0.88, 0.98)	**.036**
Cohort 1, Lunch	0–2	236	217	233	0.92 (0.87, 0.97)	**.018**	0.99 (0.93, 1.04)	.68	0.93 (0.88, 0.99)	**.044**
5.25 hours postdose	0–4	415	395	413	0.95 (0.92, 0.99)	**.033**	0.99 (0.96, 1.03)	.8	0.96 (0.92, 0.99)	.05
	0–5	495	473	492	0.96 (0.93, 0.99)	**.018**	0.99 (0.96, 1.02)	.7	0.96 (0.93, 0.99)	**.041**
**PPG incremental**	0–1	32	23	29	0.73 (0.59, 0.89)	**.014**	0.91 (0.74, 1.12)	.43	0.80 (0.65, 0.98)	.07
Cohort 1, Lunch	0–2	64	50	61	0.78 (0.64, 0.96)	.05	0.95 (0.77, 1.16)	.66	0.83 (0.67, 1.01)	.12
5.25 hours postdose	0–4	78	68	76	0.88 (0.71, 1.09)	.31	0.98 (0.79, 1.21)	.86	0.90 (0.72, 1.11)	.39
	0–5	78	69	76	0.89 (0.71, 1.11)	.36	0.97 (0.78, 1.21)	.82	0.91 (0.73, 1.14)	.49

Abbreviations: Cana, canagliflozin; LSM, geometric least square mean; LSMR, geometric LSM ratio (Sota vs. Pbo, Cana vs. Pbo, Sota vs. Cana); MMTT, mixed meal tolerance tests; Pbo, placebo; PPG, post prandial glucose; Sota, sotagliflozin. All *P* values < .05 are in bold.

^*a*^ *P* value based on null hypothesis that LSMR = 1.

After the 4.25-hour postdose lunch (Fig. 4B), incremental PPG AUC_0–1 hour_ decreased 47% (*P* < .001) with sotagliflozin and 36% (*P* = .009) with canagliflozin, and PPG AUC_0–2 hours_ decreased 31% (*P* = .033) with sotagliflozin and 30% (*P* = .044) with canagliflozin, all relative to placebo. Total PPG AUC_0–1 hour_ and AUC_0–2 hours_ were also decreased by sotagliflozin and canagliflozin relative to placebo (*P* < .05 for all comparisons).

After the 5.25-hour postdose lunch (Fig. 4C), total PPG AUC_0–5 hours_ was lower for sotagliflozin relative to both placebo (*P* = .018) and canagliflozin (*P* = .041), consistent with the R_a_O results. A post hoc analysis showed that sotagliflozin decreased incremental PPG AUC_0–1 hour_ by 27% (*P* = .014) relative to placebo and decreased total PPG AUC_0–1 hour_ and AUC_0–2 hours_ relative to placebo and to canagliflozin (*P* < .05 for all comparisons).

### Postprandial insulin and C-peptide concentrations

**Figure 5.**
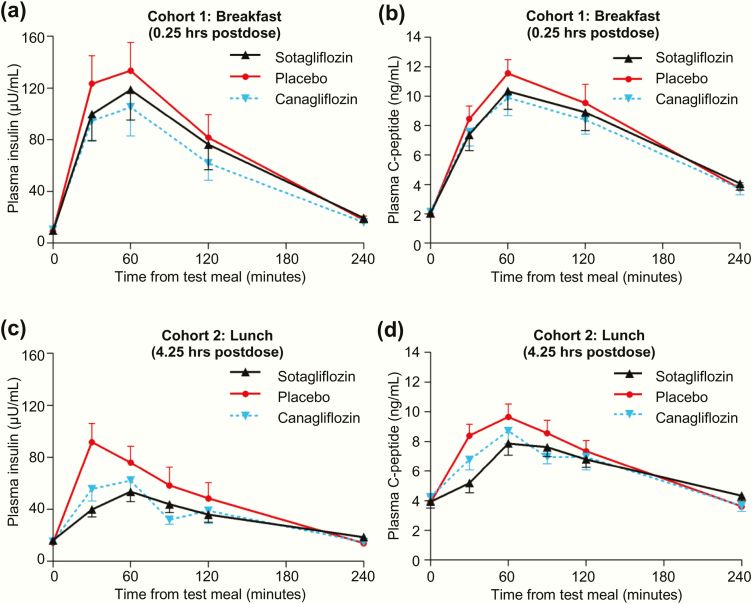
Effect of single doses of sotagliflozin or canagliflozin on (A,C) insulin concentrations and (B,D) C-peptide concentrations during the mixed meal tolerance tests. All data points are mean ± standard error of the mean.

Both sotagliflozin and canagliflozin significantly decreased incremental postprandial plasma levels of insulin and C-peptide (Fig. 5 and Table 4). After breakfast (Fig. 5A), incremental insulin AUC_0–1 hour_, AUC_0–2 hours_, and AUC_0–4 hours_ decreased 26%, 23%, and 20%, respectively, with sotagliflozin and 32%, 32%, and 31%, respectively, with canagliflozin, all *P* < .05 relative to placebo. Consistent with these results, incremental C-peptide AUC_0–1 hour_, AUC_0–2 hours_, and AUC_0–4 hours_ decreased 22%, 18%, and 13%, respectively, with sotagliflozin and 24%, 21%, and 18%, respectively, with canagliflozin, all *P* < .05 relative to placebo (Fig. 5B). During this MMTT, AUC_0–1 hour_, AUC_0–2 hours_, and AUC_0–4 hours_ for total insulin and total C-peptide also decreased with both sotagliflozin and canagliflozin, all *P* ≤ .05 relative to placebo.

**Table 4.** Total and incremental insulin and C-peptide measured following MMTTs started 0.25, 4.25 and 5.25 hours after single oral doses of sotagliflozin, canagliflozin, or placebo.

		AUC^*a*^, LSM			LSMR (90% CI of LSMR), *P*^*b*^					
Variable,MMTT	Interval (hours)	Pbo	Sota	Cana	Sota vs Pbo	*P*	Cana vs Pbo	*P*	Sota vs Cana	*P*
**Insulin total**	0–1	85	65	61	0.76 (0.62, 0.94)	**.036**	0.72 (0.58, 0.88)	**.011**	1.07 (0.87, 1.31)	.59
Cohort 1, Breakfast	0–2	180	143	129	0.79 (0.67, 0.93)	**.022**	0.71 (0.61, 0.84)	**.002**	1.11 (0.94, 1.31)	.28
0.25 hours postdose	0–4	264	218	193	0.83 (0.72, 0.95)	**.032**	0.73 (0.63, 0.84)	**.001**	1.13 (0.98, 1.30)	.16
**Insulin incremental**	0–1	76	57	52	0.74 (0.58, 0.94)	**.042**	0.68 (0.53, 0.86)	**.01**	1.10 (0.86, 1.39)	.52
Cohort 1, Breakfast	0–2	163	126	111	0.77 (0.64, 0.92)	**.023**	0.68 (0.57, 0.81)	**.002**	1.13 (0.95, 1.36)	.24
0.25 hours postdose	0–4	229	183	158	0.80 (0.68, 0.94)	**.029**	0.69 (0.59, 0.81)	**<.001**	1.16 (0.99, 1.37)	.13
**Insulin total**	0–1	62	35	44	0.56 (0.47, 0.67)	**<.001**	0.71 (0.60, 0.85)	**.003**	0.79 (0.66, 0.94)	**.033**
Cohort 2, Lunch	0–2	114	77	84	0.67 (0.57, 0.80)	**<.001**	0.73 (0.62, 0.86)	**.004**	0.92 (0.78, 1.09)	.42
4.25 hours postdose	0–4	167	128	132	0.77 (0.66, 0.89)	**.006**	0.79 (0.68, 0.92)	**.014**	0.97 (0.83, 1.12)	.72
**Insulin incremental**	0–1	48	17	25	0.36 (0.25, 0.50)	**<.001**	0.53 (0.37, 0.75)	**.005**	0.67 (0.48, 0.95)	.06
Cohort 2, Lunch	0–2	86	44	50	0.52 (0.39, 0.69)	**<.001**	0.59 (0.44, 0.78)	**.004**	0.88 (0.67, 1.17)	.46
4.25 hours postdose	0–4	111	63	69	0.57 (0.42, 0.77)	**.004**	0.63 (0.46, 0.85)	**.016**	0.90 (0.66, 1.23)	.57
**C-peptide total**	0–1	7.3	6.1	6.2	0.84 (0.73, 0.96)	**.04**	0.84 (0.74, 0.97)	**.043**	1.00 (0.87, 1.14)	.97
Cohort 1, Breakfast	0–2	17.5	15.1	14.7	0.86 (0.78, 0.96)	**.026**	0.84 (0.76, 0.94)	**.011**	1.02 (0.92, 1.14)	.69
0.25 hours postdose	0–4	30	27.1	26.2	0.90 (0.83, 0.98)	.05	0.87 (0.80, 0.95)	**.011**	1.04 (0.95, 1.13)	.46
**C-peptide incremental**	0–1	5.4	4.2	4.1	0.78 (0.65, 0.94)	**.035**	0.77 (0.64, 0.93)	**.026**	1.02 (0.84, 1.23)	.88
Cohort 1, Breakfast	0–2	13.6	11.2	10.8	0.83 (0.72, 0.94)	**.021**	0.79 (0.69, 0.90)	**.006**	1.04 (0.91, 1.19)	.59
0.25 hours postdose	0–4	22.2	19.3	18.1	0.87 (0.78, 0.97)	**.034**	0.82 (0.73, 0.91)	**.004**	1.06 (0.96, 1.18)	.33
**C-peptide total**	0–1	7.3	5.3	6.4	0.72 (0.62, 0.83)	**<.001**	0.87 (0.75, 1.01)	.13	0.82 (0.71, 0.95)	**.031**
Cohort 2, Lunch	0–2	15.6	12.6	13.6	0.81 (0.72, 0.91)	**.006**	0.87 (0.77, 0.98)	.06	0.93 (0.82, 1.04)	.28
4.25 hours postdose	0–4	26.3	23.7	23.9	0.90 (0.81, 1.00)	.09	0.91 (0.82, 1.00)	.12	0.99 (0.90, 1.10)	.9
**C-peptide incremental**	0–1	3.4	1.1	1.9	0.33 (0.20, 0.55)	**.001**	0.54 (0.33, 0.90)	**.048**	0.61 (0.37, 1.01)	.11
Cohort 2, Lunch	0–2	7.6	4.7	4.6	0.61 (0.44, 0.85)	**.018**	0.60 (0.43, 0.84)	**.015**	1.01 (0.73, 1.41)	.94
4.25 hours postdose	0–4	10.7	7.2	6.4	0.67 (0.47, 0.97)	.07	0.60 (0.42, 0.86)	*.025*	1.12 (0.78, 1.61)	*.59*

Abbreviations: Cana, canagliflozin; LSM, geometric least square mean; LSMR, geometric LSM ratio (Sota vs. Pbo, Cana vs. Pbo, Sota vs. Cana); MMTT, mixed meal tolerance tests; Pbo, placebo; Sota, sotagliflozin. All *P* values < .05 are in bold.

^*a*^Insulin AUC = h×mU/mL; C-peptide AUC = h×ng/mL.

^*b*^ *P* value based on null hypothesis that LSMR = 1.

After the 4.25-hour postdose lunch (Fig. 5C), incremental insulin AUC_0–1 hour_, AUC_0–2 hours_, and AUC_0–4 hours_ decreased 64%, 48%, and 43%, respectively, with sotagliflozin and 47%, 42%, and 37%, respectively, with canagliflozin, all *P* < .05 relative to placebo. Consistent with these results, incremental C-peptide AUC_0–1 hour_ and AUC_0–2 hours_ decreased 68% and 39%, respectively, with sotagliflozin and 44% and 39%, respectively, with canagliflozin, all *P* < .05 relative to placebo, while AUC_0–4 hours_ decreased 40% (*P* = .025) with canagliflozin, relative to placebo (Fig. 5D). During this MMTT, AUC_0–1 hour_, AUC_0–2 hours_, and AUC_0–4 hours_ for total insulin decreased significantly with both sotagliflozin and canagliflozin, while AUC_0–1 hour_ and AUC_0–2 hours_ for total C-peptide decreased significantly with sotagliflozin, all *P* < .05 relative to placebo.

### Endogenous glucose production, glucagon, and total glucose disposal rate

**Figure 6.**
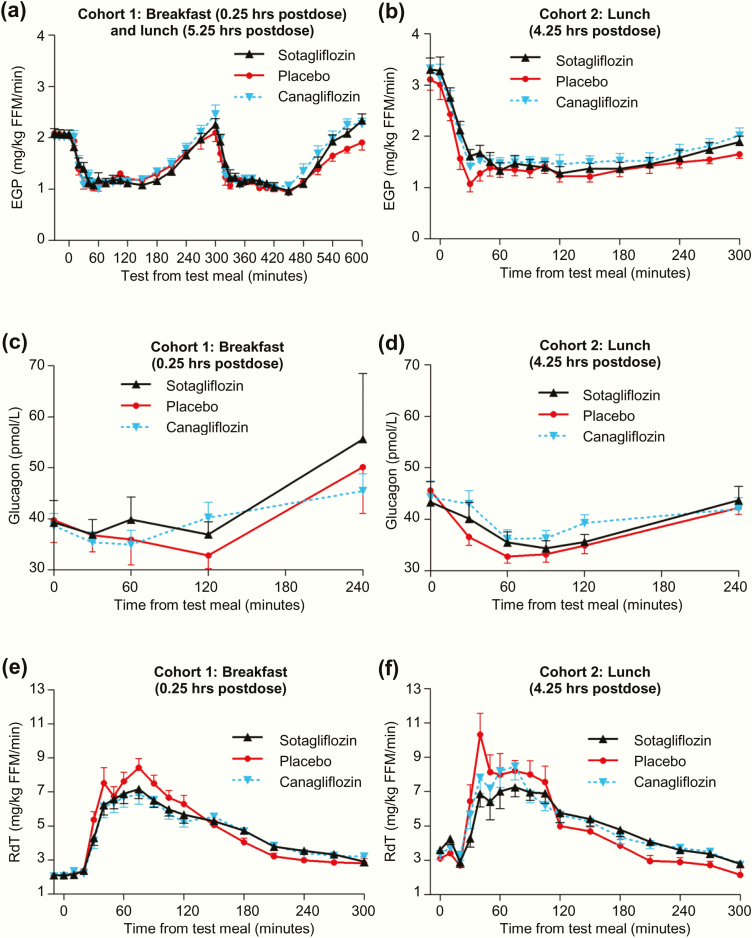
Effect of single doses of sotagliflozin or canagliflozin on (A,B) endogenous glucose production, (C,D) glucagon concentrations and (E,F) RdT during the mixed meal tolerance tests. All data points are mean ± standard error of the mean.

At baseline, the EGP rate was ~2 mg/kg fat-free mass (FFM)/min before breakfast in Cohort 1 and ~3 mg/kg FFM/min before lunch in Cohort 2, with no difference among treatment groups in either cohort. After each of the 3 MMTTs, the EGP rate was initially suppressed for 2 hours and then slowly rose through hour 5 (Fig. 6A and 6B). After breakfast (Fig. 6A), total EGP AUC_0–5 hours_ did not differ between placebo and either compound. However, after the 4.25-hour postdose lunch (Fig. 6B) total EGP AUC_0–5 hours_ increased 10% (*P* = .024) with sotagliflozin and 14% (*P* = .002) with canagliflozin, relative to placebo. The increases were similar after the 5.25-hour postdose lunch (Fig. 6A): total EGP AUC_0–5 hours_ increased 9% (*P* = .1) with sotagliflozin and 13% (*P* = .021) with canagliflozin, relative to placebo.

The effects of both compounds on glucagon mirrored their effects on EGP (Fig. 6C and 6D). Total glucagon AUC_0–4 hours_ was not increased by either compound after breakfast (Fig. 6C); however, after the 4.25-hour postdose lunch (Fig. 6D), total glucagon AUC_0–4 hours_ increased 6% (*P* = .026) with sotagliflozin and 7% (*P* = .011) with canagliflozin, relative to placebo.

The effects of both compounds on R_d_T mirrored their effects on R_a_O (Fig. 6E and 6F). After breakfast (Fig. 6E), R_d_T AUC_0–2 hours_ decreased 12% with sotagliflozin (*P* = .014) and 15% with canagliflozin (*P* < .001), while R_d_T AUC_2–5 hours_ increased 10% for both sotagliflozin (*P* < .001) and canagliflozin (*P* < .001), relative to placebo. This late compensation resulted in R_d_T AUC_0–5 hours_ that did not differ between placebo and either compound. Findings after the 4.25-hour postdose lunch showed a similar pattern (Fig. 6F).

### Intestinal peptides concentrations, gastric emptying, and urinary glucose excretion

**Figure 7.**
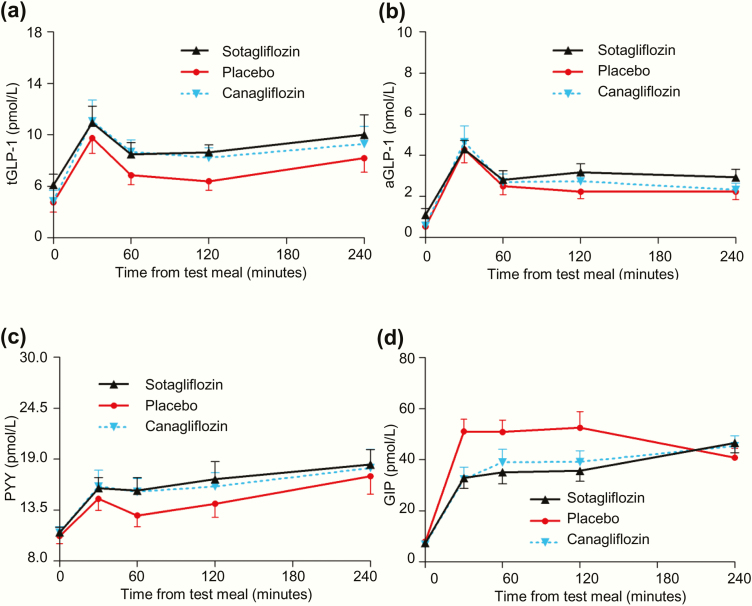
Effect of single doses of sotagliflozin and canagliflozin on intestinal peptides at the indicated times during the Cohort 1 breakfast mixed meal tolerance test: (A) tGLP1; (B) aGLP1; (C) total PYY; and (D) total gastric inhibitory polypeptide. All data points are mean ± standard error of the mean.

Both sotagliflozin and canagliflozin increased levels of GLP-1 and PYY while decreasing levels of GIP, relative to placebo. After breakfast (Fig. 7) and lunch 4.25 hours postdose (data not shown), sotagliflozin increased AUC_0–4 hours_ for total GLP-1, active GLP-1, and total PYY, while lowering AUC_0–4 hours_ for total GIP, all *P* < .05 relative to placebo. Canagliflozin increased AUC_0–4 hours_ for total GLP-1 and lowered AUC_0–4 hours_ for total GIP after breakfast, and increased AUC_0–4 hours_ for total PYY after lunch 4.25 hours postdose, all *P* < .05 relative to placebo.

Gastric emptying was determined indirectly by measuring the absorption of acetaminophen administered just after breakfast or lunch (Fig 1B). Neither sotagliflozin nor canagliflozin altered gastric emptying after breakfast; acetaminophen AUC_0–1 hour_, AUC_0–2 hours_, and AUC_0–5 hours_ were no more than 4% and 11% different from placebo for sotagliflozin and canagliflozin, respectively. After the 4.25-hour postdose lunch, acetaminophen AUC_0–2 hours_ trended down by 24% with sotagliflozin (*P* = .07) and by 23% with canagliflozin (*P* = .08), and AUC_0–5 hours_ decreased 14% by sotagliflozin (*P* = .013), all relative to placebo.

All participants provided 24-hour urine collections. For Cohort 1, the 24-hour UGE (mean ± standard deviation) was 0.9 ± 2.1 g for placebo, 42.5 ± 15.3 g for sotagliflozin (*P* < .001 vs placebo), and 73.3 ± 20.6 g for canagliflozin (*P* < .001 vs placebo and vs sotagliflozin). For Cohort 2, the 24-hour UGE was 0.5 ± 1.2 g for placebo, 44.4 ± 24.3 g for sotagliflozin (*P* < .001 vs placebo), and 60 ± 14.5 g for canagliflozin (*P* < .001 vs placebo and vs sotagliflozin).

## Discussion

In healthy adults, sotagliflozin pretreatment delayed and blunted intestinal glucose absorption during the primary endpoint MMTTs that began 0.25 and 4.25 hours after dosing, and during the exploratory MMTT that began 5.25 hours after dosing. Intestinal glucose absorption was decreased during the first 2 hours, and then increased between 2 and 5 hours of each MMTT, resulting in no net effect of sotagliflozin on total intestinal glucose absorption over that 5-hour interval. Canagliflozin pretreatment also delayed and blunted intestinal glucose absorption during the primary endpoint MMTTs that began 0.25 and 4.25 hours after dosing but had no effect during the exploratory MMTT that began 5.25 hours after dosing, consistent with published data showing that canagliflozin delayed and blunted intestinal glucose absorption during a MMTT that began 0.33 hours after dosing (23).

The effects of sotagliflozin and canagliflozin on intestinal glucose absorption are consistent with their effects on PPG. PPG was blunted after the 5.25-hour postdose lunch following sotagliflozin, but not canagliflozin, pretreatment, consistent with previous findings that canagliflozin’s effect on PPG in healthy people was present in a MMTT that began 0.5 hours postdose but lost in a MMTT that began 4.5 hours postdose (24). The blunting of PPG by sotagliflozin in healthy adults is consistent with the blunted PPG observed with sotagliflozin after glucose challenge in adults with T1D (19) and T2D (18). These PPG results were also confirmed in the T1D phase 2b dose-ranging study with prespecified mixed meal testing procedures (28) and in the larger pooled phase 3 data (17); in these studies, the reduction of PPG observed in patients with T1D who were treated with sotagliflozin was at least 2-fold greater than the reduction of fasting plasma glucose.

The decreases in intestinal glucose absorption and PPG observed with sotagliflozin were accompanied by decreases in insulin and C-peptide levels after breakfast and lunch. Decreases in insulin and C-peptide levels were also observed with canagliflozin pretreatment, consistent with previously published observations (23,24,26). Together, these findings have implications for patients with diabetes. For those with T1D, blunted PPG excursions lower A1C levels (29,30) and contribute to decreased glucose variability, particularly less time spent with blood glucose >180 mg/dL, resulting in increased TIR. Increased TIR has been linked to decreased patient distress (31) and, in the Diabetes Control and Complications Trial, to a decreased risk of microvascular complications including retinopathy and nephropathy (32). Also, based on the delay between injection of mealtime bolus insulin and its pharmacodynamic effect to lower glucose, the blunting and delay of PPG excursions should allow more robust matching of bolus insulin pharmacokinetic/pharmacodynamic properties with glycemic excursions.

For patients with T2D, blunted PPG excursions should increase TIR, which is linked to a decreased risk of retinopathy (33). Blunted PPG excursions should also decrease demand for insulin production by already compromised pancreatic β-cells, potentially slowing the progressive loss of β-cell function (34). In addition, genetic partial loss of SGLT1 function is not only associated with lower PPG, but also with reduced rates of T2D, obesity, congestive heart failure and mortality in affected individuals, suggesting that partial pharmacologic inhibition of intestinal SGLT1 by sotagliflozin may provide similar beneficial effects (35).

Published evidence suggests that the delay and blunting of intestinal glucose absorption observed here with sotagliflozin pretreatment results from local inhibition of intestinal SGLT1 (2,36–39). Although delayed gastric emptying can delay and blunt intestinal glucose absorption, this mechanism did not occur here because sotagliflozin did not alter gastric emptying (estimated by plasma acetaminophen levels) after breakfast, particularly during the first 2 hours when sotagliflozin significantly lowered R_a_O. Also, the sotagliflozin-mediated decrease in incremental PPG is likely the result of delayed and blunted intestinal glucose absorption due to SGLT1 inhibition rather than to increased rates of glucose disposal or UGE. Both sotagliflozin and canagliflozin lowered R_d_T, R_a_O, PPG, and postprandial insulin levels versus placebo over the first 2 hours postdose, providing clear evidence that the mechanism for PPG reduction involved decreased R_a_O rather than increased R_d_T, consistent with prior canagliflozin data (23). In patients with T2D, blunted PPG was observed after a canagliflozin dose of 300 mg but not 150 mg, with both doses inducing comparable UGE during a MMTT (26). The fact that canagliflozin induced greater 24-hour UGE than sotagliflozin in the present study supports the hypothesis that UGE is not the mechanism by which sotagliflozin blunts PPG excursions (2,36–40).

The similar ability of sotagliflozin and canagliflozin to decrease R_a_O during the first 2 hours of the MMTT starting 0.25 hours postdose, despite sotagliflozin being a 20-fold more potent SGLT1 inhibitor, suggests that early concentrations of both compounds in the proximal small intestine greatly exceeded their SGLT1 IC_50_ values, resulting in comparable and maximal SGLT1 inhibition in this region. The similar R_a_O pattern during MMTTs starting 0.25 hours and 4.25 hours post canagliflozin dose, and MMTTs starting 0.25, 4.25 and 5.25 hours post sotagliflozin dose, suggests that the 2 compounds inhibit SGLT1 in the proximal but not distal small intestine over the duration of these MMTTs, resulting in an early R_a_O decrease during the first 2 hours and a later increase during the last 3 hours as glucose reaches the distal small intestine where SGLT1 is not inhibited. The identical effect of sotagliflozin on R_a_O pattern during MMTTs starting 0.25 hours and 5.25 hours after a single dose in the same people on the same day strongly supports this model rather than the current model where decreased R_a_O during the first 2 hours of a breakfast MMTT indicates inhibition of intestinal SGLT1 by canagliflozin and increased R_a_O during the last 3 hours indicates loss of this inhibition (23–26).

Decreased glucose levels with SGLT2 inhibitors are offset by increased EGP, which is observed after single or multiple doses of dapagliflozin (41), empagliflozin (42), and canagliflozin (23,43). In each case, increased EGP was accompanied by increased glucagon and decreased insulin levels. The same pattern was observed in this study after single doses of canagliflozin or sotagliflozin, providing further evidence that increases in EGP and glucagon are class effects. The increased EGP is likely secondary to inhibition of SGLT2 rather than SGLT1 because it is induced by all 4 compounds. The mechanism behind the increased EGP probably involves increased UGE, the most striking physiological effect of SGLT2 inhibition, but appears to be independent of changes in levels of pancreatic hormones, because adding liraglutide to canagliflozin pretreatment prevented the canagliflozin-mediated changes in glucagon and insulin levels but did not block the increases in EGP or UGE (43).

In contrast to our findings, data on self-monitored blood glucose (SMBG) from the Phase 3 inTandem1 study presented at the US Food and Drug Administration Endocrinologic and Metabolic Drugs Advisory Committee Meeting on sotagliflozin (44) suggested that sotagliflozin had little effect on PPG excursions. However, we believe that an appropriate PPG evaluation should include a comparison to baseline under standard meal and insulin administration conditions because the effect of sotagliflozin on PPG would then be separated from the effect of prandial insulin on PPG since prandial insulin remains constant. The SMBG analysis cited above did not include these conditions (9). For that SMBG analysis, SMBG targets, including PPG, were assessed by an independent insulin dose monitoring committee that provided feedback to investigators on insulin dose adjustment if standard-of-care glucose targets were not met (9). Under these intensive insulin therapy conditions, similar PPG glycemic excursions in placebo and sotagliflozin study arms would be expected, and would reflect appropriate insulin adjustments to meet PPG glycemic targets. The fact that this was observed (44) is a demonstration that intensive insulin therapy was achieved during the study. These similar PPG excursions observed in placebo and sotagliflozin study arms were accompanied by decreased bolus insulin dosing in the sotagliflozin arms relative to placebo (9); thus, the PPG excursions were influenced by 2 drugs: sotagliflozin and insulin. When PPG was assessed under conditions of standard meal and insulin administration in phase 2 and 3 studies of sotagliflozin in people with T1D, which represents the effects of sotagliflozin only on PPG, statistically significant and clinically meaningful reductions in PPG were indeed observed with sotagliflozin (17,28), consistent with the glucose absorption data presented here.

Study limitations included the small number of participants, the absence of MMTTs conducted more than 5.25 hours after treatment and the post hoc analysis of some R_a_O and PPG data. Also, because adjustments for multiple comparisons were not performed due to the exploratory nature of the study, nominal *P* values and confidence intervals were provided as descriptive statistical summaries of the statistical data to guide inferences, rather than as rigorous proof of the conclusions. Accordingly, the results are hypothesis generating for the effects of sotagliflozin in patients with diabetes.

In conclusion, sotagliflozin blunted and delayed intestinal glucose absorption in healthy adults, resulting in lower PPG and insulin levels. The underlying mechanism was likely prolonged local inhibition of intestinal SGLT1 that persisted for at least 5 hours after sotagliflozin dosing. The sotagliflozin-mediated decrease in GIP and increases in GLP-1 and PYY levels after oral challenge with a glucose-containing meal are consistent with local inhibition of intestinal SGLT1.

The datasets generated during and/or analyzed during the current study are not publicly available but are available from the corresponding author on reasonable request.

## Abbreviations

A1C: glycated hemoglobin
AUC: area under the curve
EGP: endogenous glucose production
FFM: fat-free mass
GIP: gastric inhibitory polypeptide
GLP: glucagon-like peptide
IC50: median inhibitory concentration
MMTT: mixed meal tolerance test
SGLT: sodium–glucose cotransporter
PPG: postprandial glucose
PYY: peptide YY
RaO: rate of appearance of oral glucose
RaT: rate of appearance of total glucose in blood
RdT: total glucose disposal
T1D: type 1 diabetes
T2D: type 2 diabetes
TIR: time in range
UGE: urinary glucose excretion.

## Additional Information

***Disclosure Summary:*** D.R.P. is employed by and holds stock options in Lexicon Pharmaceuticals, Inc. B.Z. is employed by Regeneron Pharmaceuticals, Inc and was previously employed by and consulted for Lexicon Pharmaceuticals, Inc. L.M. is employed by ProSciento, Inc. C.B. is employed by ProSciento, Inc. M.H is employed by and has equity interests (including stock options) in ProSciento, Inc. S.T. is a previous employee of Kinemed, and is currently employed by Pliant Therapeutics. M.H. has no conflicts to declare. P.B. is employed by and holds stock options in Lexicon Pharmaceuticals, Inc. P.S. is a previous employee of and has equity interests (including stock options) in Lexicon Pharmaceuticals, Inc and is currently employed by Metavant Sciences, Ltd. P.L. is employed by Lexicon Pharmaceuticals, Inc.

***Data Availability:*** The datasets generated during and/or analyzed during the current study are not publicly available but are available from the corresponding author on reasonable request.
